# Supplementary figures and images for: Numerous recursive sites contribute to accuracy of splicing in long introns in flies
Source: PLoS Genet. 2018 Aug 27;14(8):e1007588. doi: 10.1371/journal.pgen.1007588 (PMC6110457; doi:10.1371/journal.pgen.1007588)

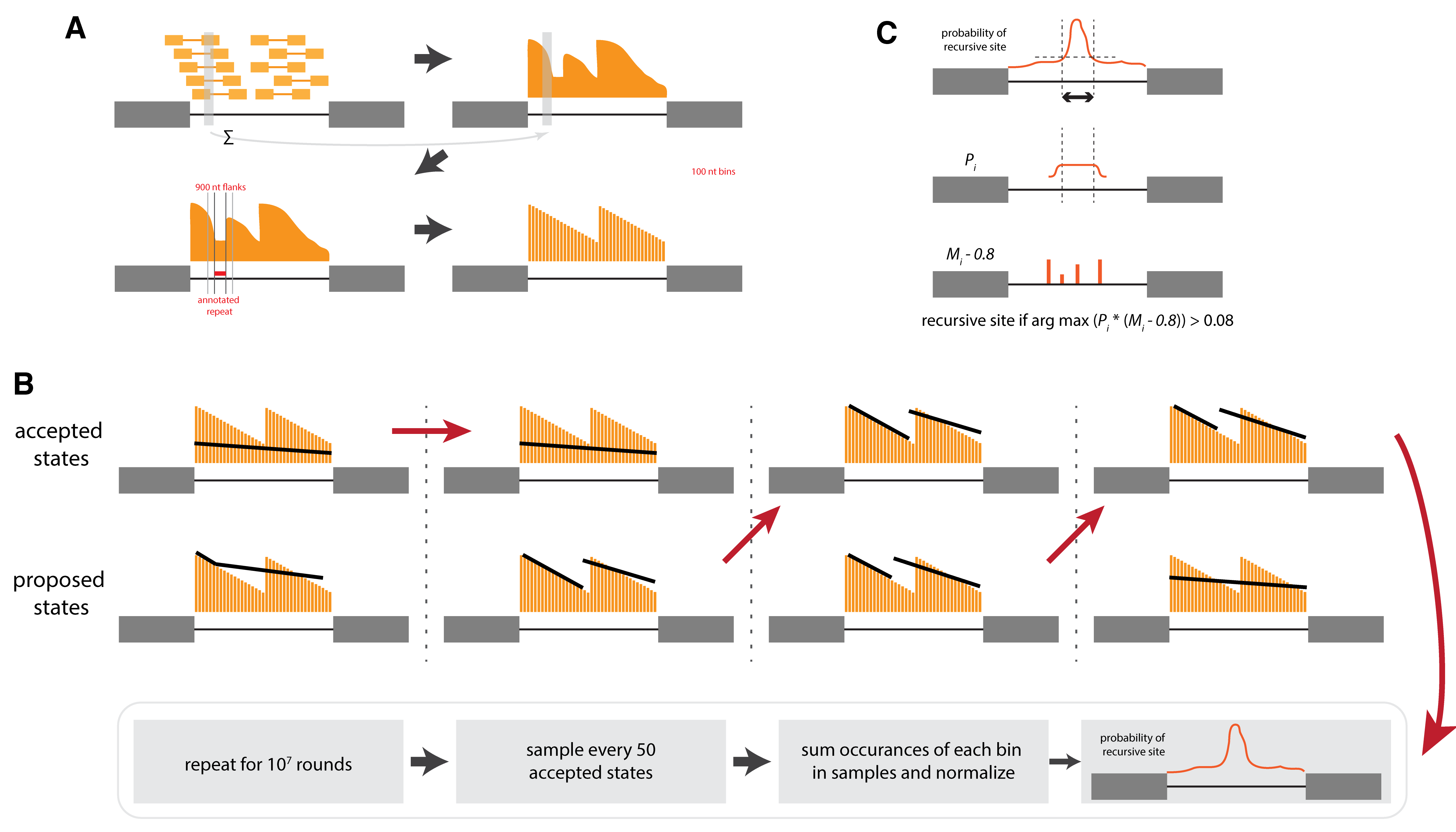

Supplement: S1 Fig — (A) RNA-seq pre-processing steps to convert reads into an array of read densities: (1) summing the read coverage for each base-pair (top) and (2) replacing the read counts in annotated repeat regions and 100 flanking nt with median read density in 900 nt flanking regions (bottom) (B) MCMC algorithm infers probability that each position in intron is a recursive splice site, where upon entering each round with a previously accepted state, this state is perturbed to propose a new state and the new state is either accepted or rejected. The procedure is performed over 107 rounds, with sampling every 50 rounds to obtain a probability that each base pair is a recursive site. (C) Sequence information is used in conjunction with MCMC-inferred probabilities to predict recursive sites. (TIF) [file pgen.1007588.s001.tif]

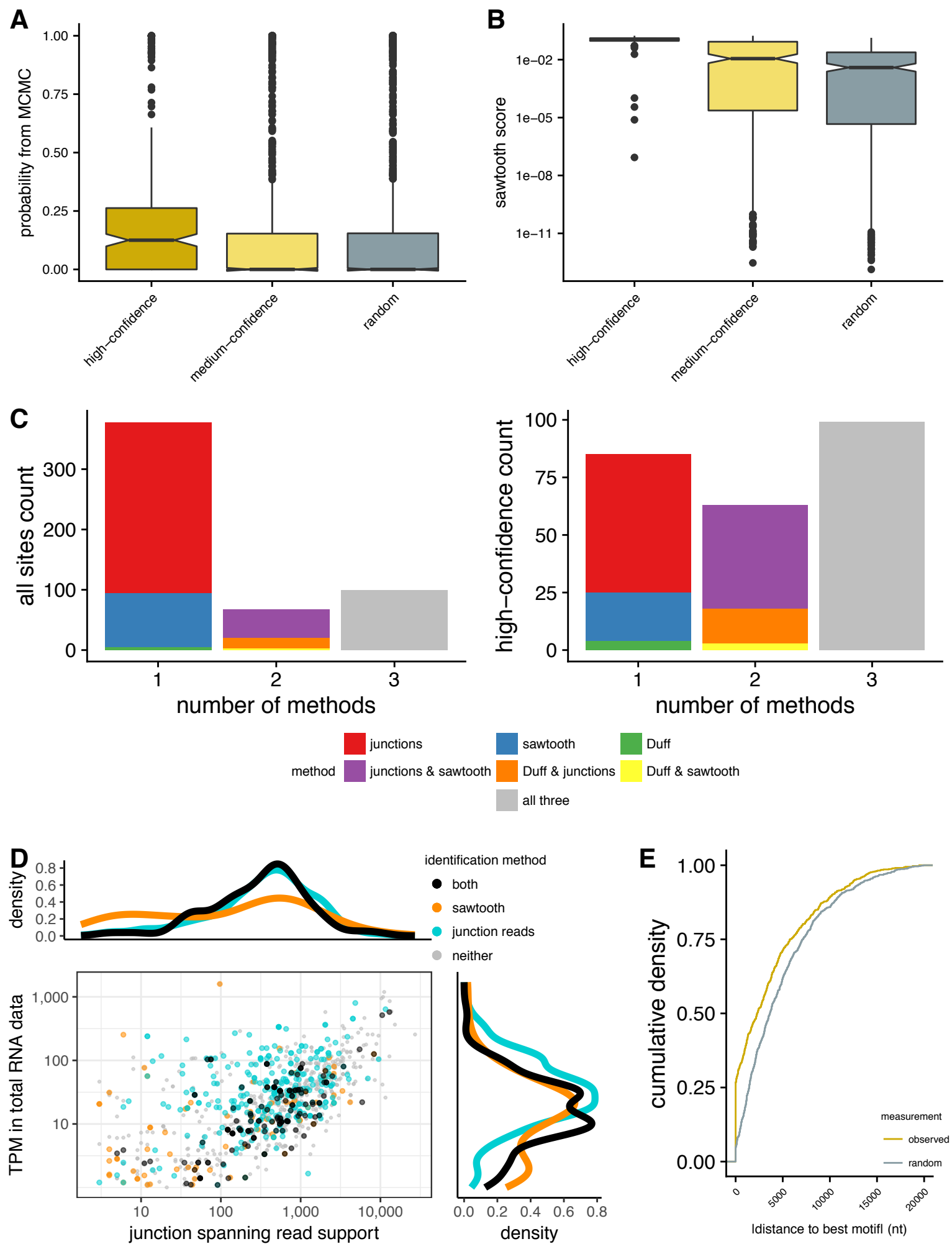

Supplement: S2 Fig — (A) The probability derived from the sawtooth MCMC model of a site being a recursive site for the final set of recursive sites (light orange), all sites with minimal support from any method (dark orange), and random sites placed down in the same introns (grey). (B) The sawtooth score (see Methods) for the final set of recursive sites (light orange), all sites with minimal support from any method (dark orange), and random sites place down in the same introns (grey). (C) Number of recursive sites (left) and high-confidence sites (right) identified by one of multiple identification pipelines, with the majority of recursive sites identified by both junction reads and sawtooth scores, as well as present in the Duff et al. dataset. (D) The gene expression levels of genes with recursive introns (TPM, y-axis) relative to the junction spanning read support for each recursive intron (read count, x-axis), showing the varying power to identify recursive sites with the sawtooth recursive method (orange), junction-spanning reads alone (blue), or both methods (black). (E) The cumulative distribution of distances between the recursive site identified with the sawtooth recursive method and the best matching recursive motif (orange) and random sites placed down in the same introns (grey) are significantly different. (PDF) [file pgen.1007588.s002.pdf]

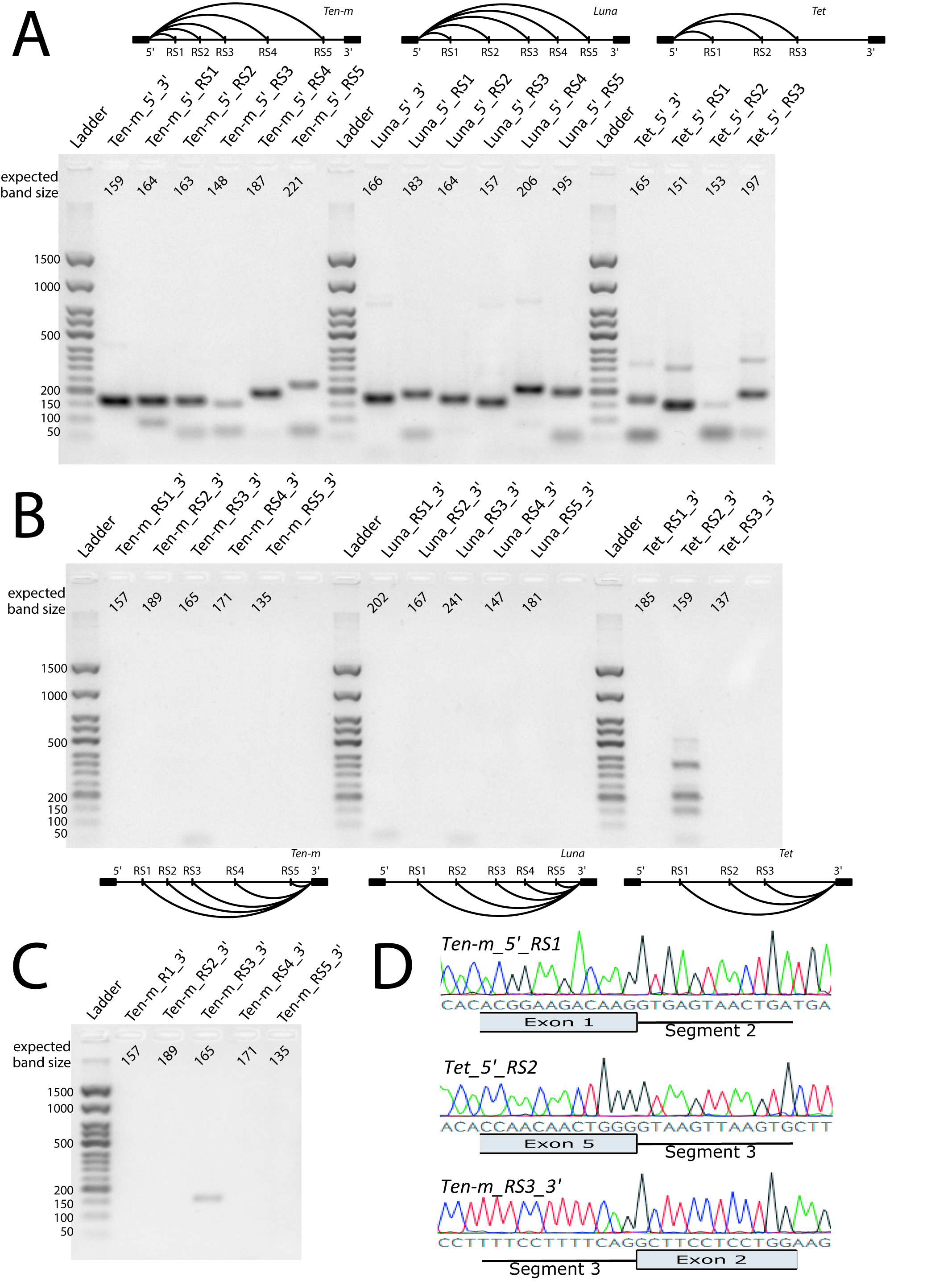

Supplement: S3 Fig — PCR products from recursive segments anchored to intronic 5' splice sites (A) and intronic 3' splice sites (B), for recursively spliced introns from three genes: Ten-m (left), Luna (middle), and Tet (right). PCR was performed on first-strand cDNA from nascent RNA from Drosophila S2 cells isolated after 5 minutes of labeling with 4sU and bands were visualized on a 1.5% agarose gel with a 50bp ladder. Schematics indicate the junctions across which the amplicons were designed. Junction boundaries were confirmed by sequencing for lanes 3, 5, 17, 18, and 19 in (A). (C) PCR products across recursive segments and intronic 3' splice sites from first-strand cDNA from steady-state RNA for the Ten-m recursively spliced intron. The junction boundary was confirmed by sequencing for lane 4. (D) Representative sequence traces confirming the junction boundaries for two novel 5'-RS recursive splicing events (top and middle) and one RS-3' event (bottom). Peaks delineate specific nucleotides, including A (green), C (blue), G (black), and U (indicated by a T, red). For each of these events, the full band was sequenced (164nt, 153nt, ad 159nt from top to bottom respective), however only 30nt around the junction is shown here for visualization purposes. (TIF) [file pgen.1007588.s003.tif]

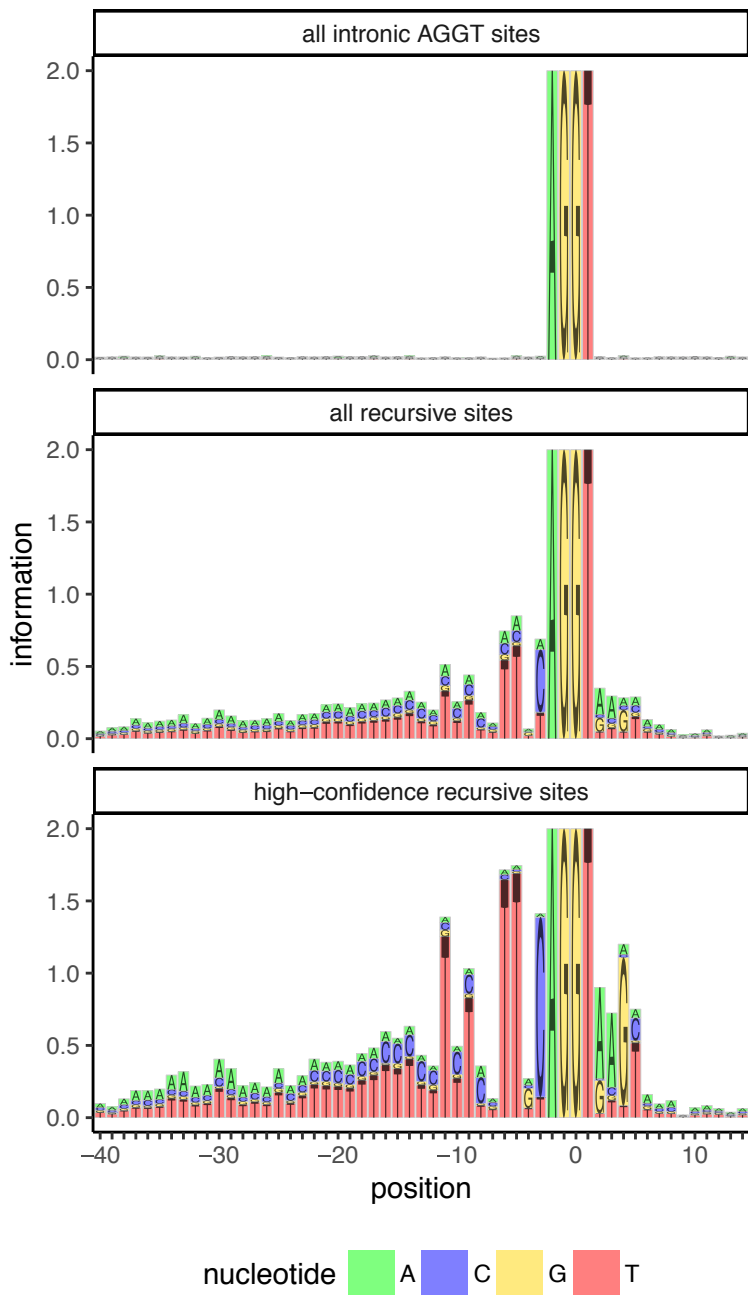

Supplement: S4 Fig — Sequence logo for all intronic AG|GT sites (top), medium-confidence recursive sites (middle) and high-confidence recursive sites (bottom). (PDF) [file pgen.1007588.s004.pdf]

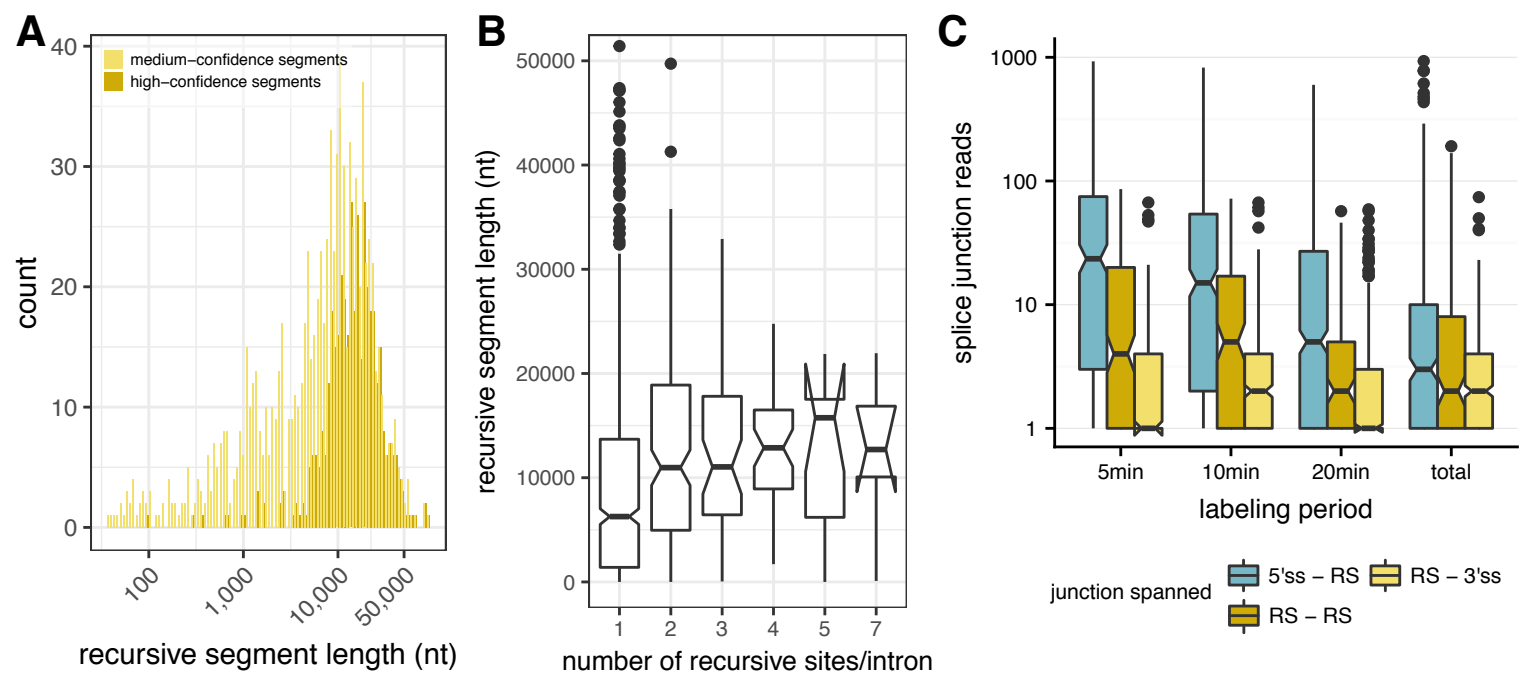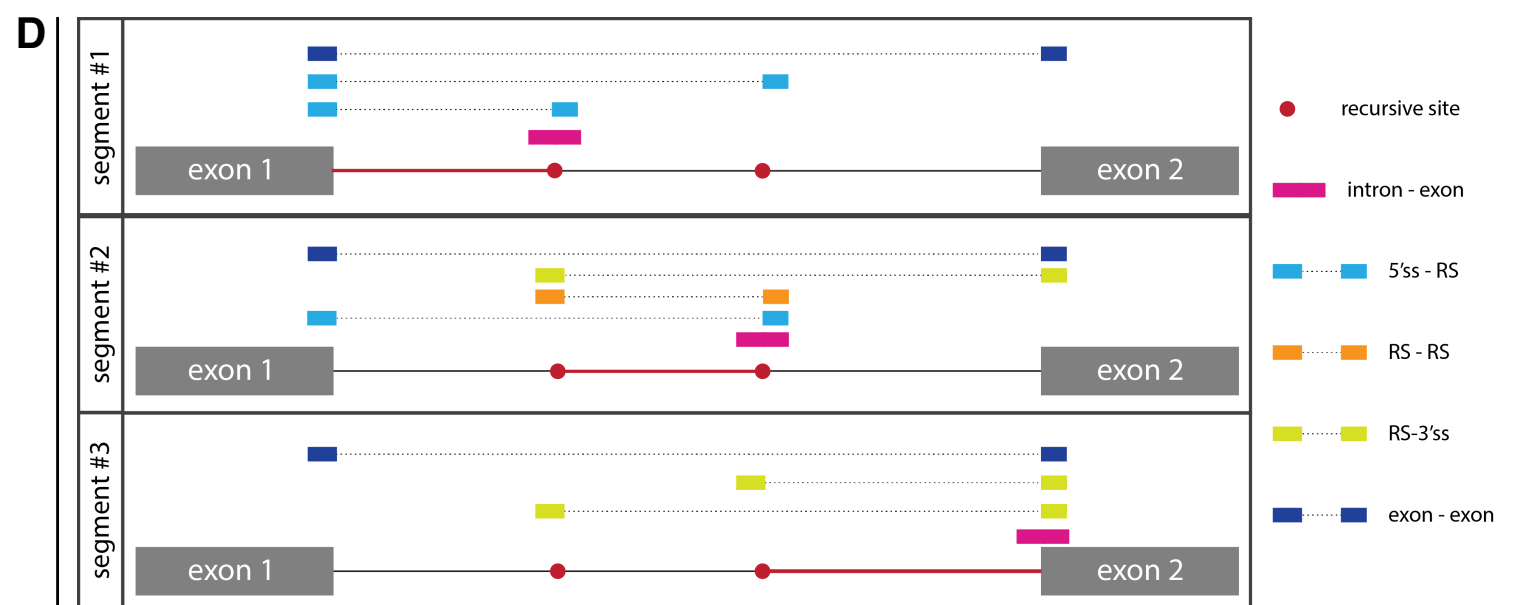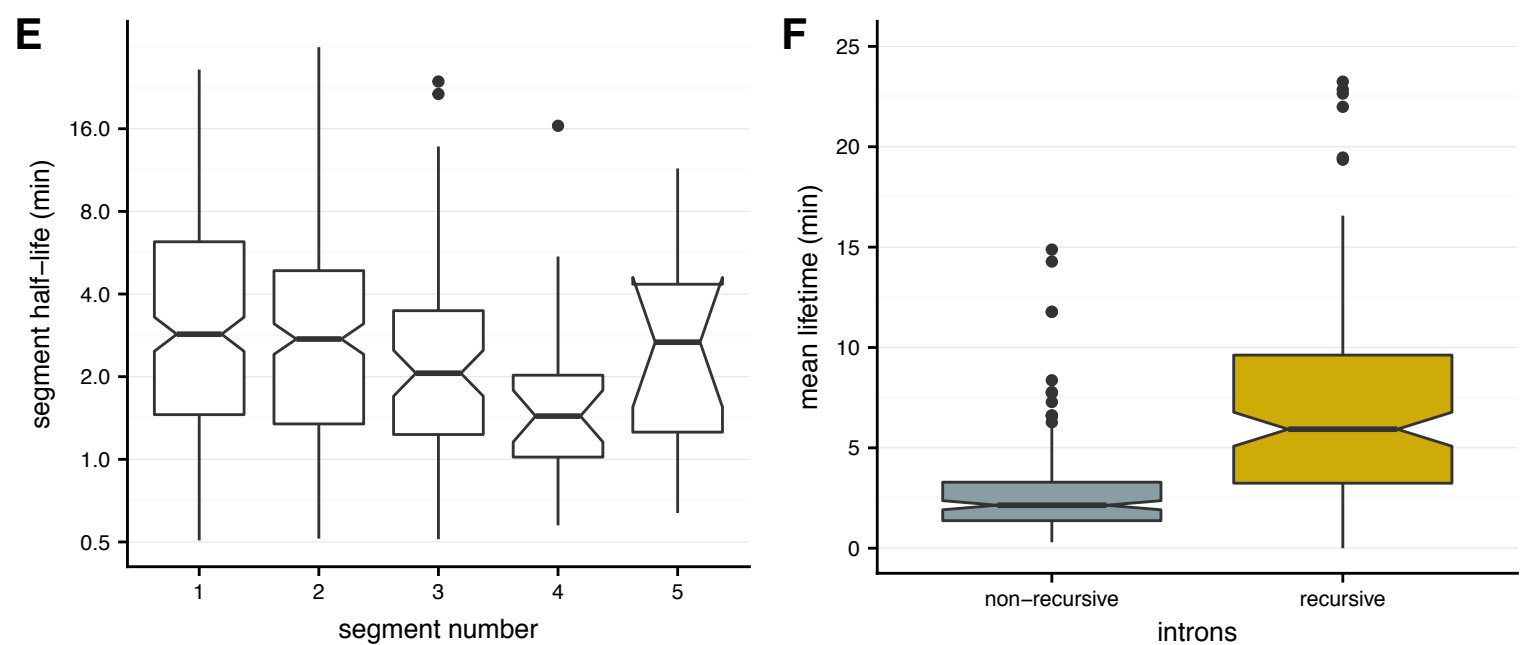

Supplement: S5 Fig — (A) Distribution of lengths of recursive segments (nucleotides, x-axis) for medium-confidence recursive segments (yellow) and high-confidence recursive segments (gold). (B) Recursive segment length distributions (nt, y-axis) for introns with varying numbers of recursive sites (x-axis). (C) The number of splice junction reads (y-axis) spanning a 5' splice site and recursive site (blue), two recursive sites (gold), and a recursive site and 3' splice site (yellow) across the labeling periods (x-axis). (D) Junction reads used to estimate splicing half-lives for recursive segments (red lines), centered on 3' recursive sites (red dots) for each segment. Incomplete splicing is estimated from intron-exon junction reads (pink bars). Completed splicing is estimated from a sum across split-junction reads between the 5' splice site and recursive site (light blue bars), two recursive sites (orange bars), a recursive site and the 3' splice site (yellow bars), and the 5' splice site and 3' splice site (exon-exon read, dark blue bars). Each segment’s splicing is informed by different types of junction reads dependent on the position in the intron, as drawn for an intron with three recursive segments. (E) Splicing half-lives (y-axis) for recursive segments with varying positions across the intron (x-axis), where on average, all segments in an intron tend to be spliced out at similar rates. (F) The distribution of mean life-times (y-axis) for recursively spliced introns (estimated by the maximum of exponentials from constituent recursive segment splicing rates, gold) relative to non-recursive introns chosen to match the length of the recursive introns (grey). (PDF) [file pgen.1007588.s005.pdf]

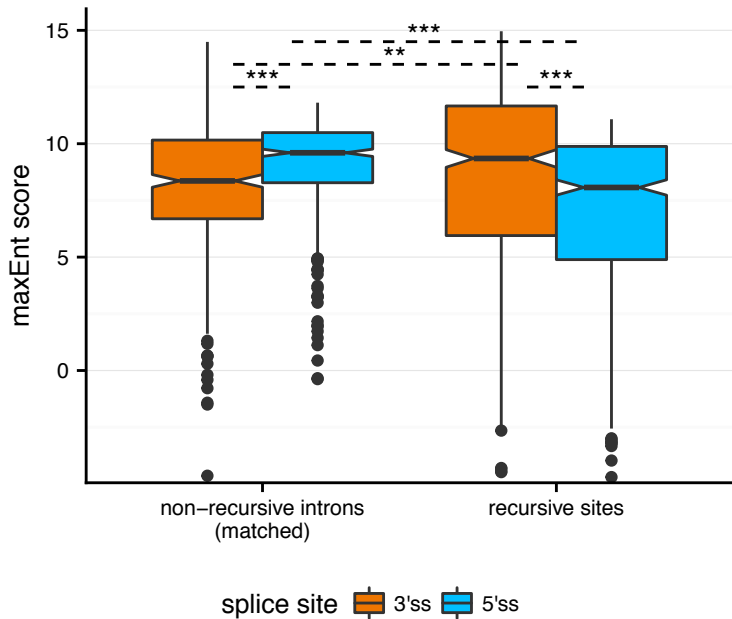

Supplement: S6 Fig — Distribution of splice site strengths (maxEnt score, y-axis) across both 3' splice sites (orange) and 5' splice sites (blue) for recursive sites (right) and non-recursive introns matched for intron length (left). Significance is indicated such that **: P < 0.01 and ***: P < 0.001, with a Mann-Whitney U test. (PDF) [file pgen.1007588.s006.pdf]
